# A novel strategy to downstage breast cancer: impact of a phone helpline

**DOI:** 10.3332/ecancer.2023.1637

**Published:** 2023-11-21

**Authors:** Neetha Mary Kurian, Jeffrey Mathew Boby, Somannair Suneesh, Sumit Datta, Aju Mathew

**Affiliations:** MOSC Medical College, Kolenchery 682311, Kerala, India

**Keywords:** breast cancer, screening, India, telephone helpline, downstage, cancer

## Abstract

Breast cancer incidence rates in India are rising. The majority of breast cancers are still diagnosed in later stages. There is also a burden of neglected cancers in India, where patients neglect their symptoms due to fear, ignorance, financial insecurity and lack of access to medical care. This results in greater morbidity and mortality from breast cancer. Systematic screening programs have been tested in an Indian setting, with limited success. An effective strategy to downstage breast cancer is an area of unmet need. We aimed to explore the effectiveness of an anonymous nurse-led telephone helpline in identifying patients with possible breast malignancies and to encourage them to seek healthcare. We created a telephone helpline system by training junior public health nurses (JPHNs) to provide counselling to women who may call with breast-related symptoms. We then created a short video message on the initiative and disseminated it using social media platforms. During the 1-year study period, 434 calls were received from individuals who reported having some breast symptoms. Among them, 28% (122 callers) had never consulted a doctor for their symptoms. 78 callers consulted a nearby doctor upon the advice of the JPHN. Among them, 14 callers (18%) were advised by the doctor to undergo investigations to rule out malignancy, while 64 (82%) of them were found to have some benign/normal breast conditions. 3 (21%) out of the 14 patients who underwent further evaluation were eventually diagnosed with breast cancer. Our study provides evidence that an anonymous nurse-led telephone helpline can be an effective strategy to reduce the incidence of neglected breast cancers and downstage the diagnoses.

## Introduction

Breast cancer is the most common cancer among women worldwide constituting around 25% of all cases [1]. India has a rising incidence of these cancers with 180,000 cases being reported in 2020 [2, 3]. The prevalence of triple-negative breast cancer (TNBC), an aggressive form of the disease with a poor prognosis, is particularly high in India [4, 5]. A meta-analysis by Sandhu *et al* [4] reported the overall combined prevalence of TNBC as 31% in India compared to around 12% in the United States [6]. Systematic screening may not be relevant in India as highly aggressive cancers such as TNBC and HER2-amplified cancers are often missed during the interval period between screening tests [7]. Interval cancers refer to those cases that develop between the recommended screening periods and hence cannot be picked up early by regular mammograms. TNBC is especially prone to this phenomenon [5].

Additionally, there is a burden of neglected cancers in India, where patients neglect their symptoms due to fear, ignorance, financial insecurity and lack of access to medical care. As a result, the majority of breast cancers in India are diagnosed in the stages of locally advanced or metastatic disease. This unfavourable epidemiological pattern results in greater morbidity and mortality from breast cancer.

In an effort to identify strategies to reduce the incidence of neglected breast cancers, we aimed to explore the effectiveness of an anonymous nurse-led telephone helpline in identifying patients with possible breast malignancies. The objective of this study was to determine whether the implementation of such a helpline could downstage breast cancer diagnoses in India, particularly among those who may not have access to regular screening programs.

By implementing an anonymous nurse-led telephone helpline, patients can seek advice on breast health concerns and receive guidance on the next steps for diagnosis and treatment. The anonymity of the helpline may help to reduce the fear and stigma associated with breast cancer, which may encourage patients to seek medical attention sooner. Furthermore, the nurse-led approach may help provide accessible and affordable healthcare to patients.

In this study, we aimed to evaluate the impact of the anonymous nurse-led telephone helpline in the state of Kerala, India. We hypothesize that the implementation of this helpline will lead to patients, who otherwise had not seen a doctor or a nurse to discuss their breast-related symptoms, being referred for diagnostic tests to rule out breast cancer.

## Methods

We created a telephone helpline system by training five junior public health nurses (JPHNs) to provide counselling to women who may call with breast-related symptoms (Oppam helpline; Oppam is a Malayalam word that means ‘with you’). The JPHNs involved in attending the phone calls were given training on various aspects of breast cancer, prior to the launch of the program. Frequently asked questions and their answers were prepared by the first author (faculty of the Community Medicine Department) in the local language, referring to data and documents from the WHO site. Four sessions on how to attend the calls, advice to be given to the callers on common questions, counselling the callers to tide over the difficult situation, and motivating them to consult a doctor were taken by the first author. Further training through two sessions based on NCCN Guidelines for Patients: Invasive Breast Cancer, 2020 was carried out by another Professor from the Community Medicine department. Topics like benign and malignant breast conditions and surgical management of breast cancer were also covered in two classes conducted by a Professor of Surgery. The pathological aspects of breast conditions were taught in two separate discussions headed by a professor from the pathology department of the institution. The JPHNs were finally asked to summarize and discuss all of the classes attended and each of them were evaluated based on their response. In addition to being trained on how to provide counselling and support to callers who may be anxious or afraid, they were also instructed to refer patients to a physician or surgeon if the symptoms seemed suspicious of breast cancer.

We then developed a 4-minute-long video-based message cautioning women about neglecting their breast symptoms and counselling them on the availability of the helpline to anonymously discuss their concerns. The video was designed to be informative and easy to understand, with the aim of encouraging women to seek help if they had any concerns about their breast health. The video was then released through various social media platforms like Facebook and YouTube pages of Malankara Orthodox Syrian Church Medical College and the hotline was publicized during several health awareness sessions conducted in neighbouring localities of the hospital. Posters with the hotline number were displayed in five rural outreach health facilities run by the Department of Community Medicine as well as government anganwadies situated near these health centres. We also used the contact list of the principal investigator to disseminate the message and encouraged the recipients to send it to their contacts – a snowballing strategy to share the video with a wider group of individuals.

Six months from the initial launch, we sent one more video-based message to remind women about the importance of breast health and the availability of the helpline. The second message also encouraged women to seek help if they had any concerns about their breast health.

We then gathered data on all calls received in the helpline over a period of 1 year. Information on the number of calls received and their phone numbers, the nature of the concerns reported by patients, and the outcomes of the calls, including referrals for further evaluation and treatment, were collected. The outcome of each call was also followed up 1 week later. Each JPHN would submit a detailed summary of all the calls received in an earlier agreed-upon prescribed format to the first author at the end of every month. This was then entered into Microsoft Excel sheets and analysed. To analyse the data, we used descriptive statistics to report the number of calls received and the nature of the concerns reported.

## Results

During the 1-year study period, a total of 750 calls were received on the telephone helpline. The average number of calls per day was 2.

Of the total calls received, 58% (434 calls) were from individuals who reported having some breast symptoms. Among them, 72% (312 callers) had already consulted a doctor, while 28% (122 callers) had never consulted a doctor for their symptoms.

Of the 122 callers who had not previously consulted a doctor, 78 callers (64%) consulted a nearby doctor upon the advice of the JPHN who attended to their call. Among them, 14 callers (18%) were advised by the doctor to undergo investigations to rule out malignancy, while 64 (82%) of them were found to have some benign/normal breast conditions. 14 patients who underwent further evaluation were followed up and 3 (21%) of them were eventually diagnosed with breast cancer.

We also received 7% (49 calls) of the total calls from individuals who had symptoms not related to breasts. Furthermore, the helpline received 250 calls (33%) from individuals who called to enquire about OPPAM, mostly seeking financial assistance to manage their self-reported cancer.

## Discussion

The rising incidence of breast cancer among the Indian population calls for immediate measures to curb this public health crisis. Effective screening and early diagnosis are the two common strategies employed to reduce breast cancer-associated mortality. Screening refers to the identification of cancers in a healthy population before patients develop any signs or symptoms of the disease. In contrast, early diagnosis is characterized by timely detection of cancers in their preliminary stages thus improving the chances of survival. The differences between early diagnosis and screening are further detailed in Table 1 [8].

Population-wide routine screening with mammograms similar to Western countries is unlikely to be effective in low-middle-income nations like India. This is due to a multitude of reasons. Firstly, breast cancer develops at a much younger age, a decade earlier, among Indians compared to other high-income countries [9]. Younger age is associated with denser breast tissue reducing the sensitivity of mammograms and increasing the risk of false positive results [10]. Secondly, establishing a nationwide screening program would require significant improvement in infrastructure as well as diagnostic facilities to make imaging accessible to all [7]. It would also require major investment in the training of essential health care professionals as well in health care delivery. This might prove to be economically unviable for developing nations like India which has limited resources as well as other pressing health concerns to address. Lastly, the larger proportion of TNBC among the Indian population makes them vulnerable to interval cancers [11]. Rapidly advancing nature of TNBC makes them harder to detect with routine screening and hence regular mammograms would be ineffective in bringing down the mortality. Overdiagnosis resulting in unwanted tests and treatment, false-negative results, and radiation exposure are some other caveats associated with mammography [12]. 

Standardized clinical breast examination (CBE) appears to be a cost-effective alternative to mammograms. A randomized controlled trial evaluating the efficacy of CBE in India reported that CBE resulted in earlier diagnosis and improved survival but failed to decrease breast cancer-associated mortality as well as the incidence of advanced breast cancers [13]. A similar study with a follow-up period of 20 years showed that CBE was successful in downstaging the disease and brought about a 30% significant reduction in mortality in women older than 50 years [14]. However, no such improvement in mortality was observed in the younger population. Even though CBE was successful in downstaging breast cancer the human resource investment required for its successful implementation makes it cumbersome and difficult to implement on a wider scale. We believe our helpline is a more feasible alternative. The need for minimum resources and scalability of this approach may help it to reach a large population of India with much less effort. Although other modalities of screening like ultrasonography and magnetic resonance imaging exist, multiple challenges pertaining to their efficacy and affordability prevent them from being integrated into routine clinical practice.

Early diagnosis is a much more practical approach to bring down breast cancer mortality. Detecting malignancies early will reduce tumour size at initial diagnosis and prevent lymph node spread as well as distant metastasis, eventually translating into better patient outcomes [15]. Prompt diagnosis and management are also associated with milder adverse effects from treatment and reduced cost of cancer care [16]. This is especially important in India as around 60% of newly diagnosed cases present at advanced stages of the disease [17]. Awareness about the common manifestation of cancer as well as the need for timely evaluation among the public, adequate access to well-trained health professionals, and affordable imaging modalities are prerequisites for early diagnosis.

Lack of knowledge about the common signs and symptoms of breast cancer continues to be a major hurdle in preventing prompt diagnosis [18–20]. This is especially true in rural India, a study from a low socio-economic community reported that only 50% of the sample population were aware of symptoms of the disease and less than 20% knew about its risk factors [18]. Apart from the general public, health workers also lag in awareness. A study showed that only 50% of nurses had sufficient knowledge of risk factors and screening methods for breast cancers [21]. Hence, educating the public as well as health professionals on the common presentations and risk factors of breast malignancies would go a long way in ensuring its early diagnosis.

Financial burdens also act as barriers to seeking care for cancer [22]. The high cost of diagnosis and treatment as well as the cost associated with travel were cited as major reasons for skipping treatment by 50% of the studied population [19, 20]. Sociocultural factors like lack of time, fear of being ostracized by society, apprehension of a cancer diagnosis as well as its management and fear of not being able to meet familial responsibilities also prevent early diagnosis [19, 20, 23]. Lack of access to hospitals and unavailability of female doctors are some other logistical constraints preventing prompt cancer care delivery [20]. Hence women often tend to put off hospital visits until the symptoms become unbearable or significantly limit their capacity to work. Most breast lumps being painless in the beginning may contribute to this delay. Moreover, they may also initially consult a quack or unregistered medical practitioner further delaying the diagnosis. This is especially common among the rural population [24].

Our study shows that the implementation of an anonymous nurse-led telephone helpline can reduce the number of neglected breast cancers diagnosed in India, particularly among those who may not have access to regular screening programs. The anonymity of the helpline may help to reduce the fear and stigma associated with breast cancer, which may encourage patients to seek medical attention sooner. The nurse-led approach may also help to improve the accessibility of healthcare to patients. The intervention was also inexpensive to implement.

However, our study has some limitations. First, the study was conducted over a relatively short period of time, and a longer follow-up period is needed to evaluate the long-term impact of the helpline on breast cancer diagnosis rates. Second, the study was conducted in a specific population, and the generalizability of the findings to other populations may be limited. Third, we were unable to obtain written consent from any of the callers. However, consent can be considered to be implied especially since participants themselves are initiating the call. It would be impractical to obtain written consent as this put the anonymity of the helpline into question, thus decreasing participation. Written consent would also be difficult to implement in such a mass contact program with a unique study design. Fourth, the reach of the helpline was restricted to only those who were recipients of the video-based message. Fifth, we did not compare the effectiveness of the anonymous nurse-led telephone helpline to other screening or diagnostic methods. Finally, we did not collect data on the socioeconomic status of the patients who used the helpline, which may be an important factor in determining the impact of the helpline [22].

We hypothesize that a more organized and disseminated government-sponsored phone helpline with repeated public health messaging through mass media and social media tools would make a meaningful impact on our society. Such an intervention can be widely adapted across geographical boundaries and socioeconomic divisions. It may be more cost-effective and easily scalable than CBE.

## Conclusion

In conclusion, our study provides evidence that an anonymous nurse-led telephone helpline can be a strategy to reduce the incidence of neglected breast cancers. Eventually, we believe such a strategy will downstage breast cancer diagnosis. Further research is needed to evaluate the long-term impact of the helpline and to determine the optimal approach for implementing such a helpline in other populations.

## Conflicts of interest

We declare no competing interests.

## Funding

MOSC Medical College Kolenchery funded the project and provided the JPHN staff.

## Author contributions

NK supervised the project and analysed the data. AM designed the project and the study design, supervised the project, analysed the data, and wrote the first draft of the manuscript. JB analysed the data and wrote the manuscript in various stages of revision. SS analysed the data and assisted with the project. SD supervised the project and analysed the data. All authors made substantial contributions to the manuscript and approved the final version.

## Figures and Tables

**Table 1.** Strategies to downstage cancer: early diagnosis versus screening.

Early diagnosis	Screening
Performed in symptomatic patients	Performed in a healthy, asymptomatic target population
Requires less infrastructure and investments	Requires more infrastructure and investment
Facilitated by improved awareness about the disease among the public and health community as well as ready access to diagnostic and treatment facilities	Facilitated by screening modalities that can effectively detect latent cancer along with resources for confirming the diagnoses and providing follow-up treatment.
Not associated with over-diagnosis or false results	Can lead to false positive and negative results as well as overdiagnosis
Associated with improved patient outcomes in all types of cancers	Performed in only selected cancers like breast, colorectal, and cervical cancer
Biopsy, blood tests, etc., can be used for early diagnosis	Mammograms, pap smears, etc can be used for screening
